# Dietary Egg Sphingomyelin Prevents Aortic Root Plaque Accumulation in Apolipoprotein-E Knockout Mice

**DOI:** 10.3390/nu11051124

**Published:** 2019-05-21

**Authors:** Courtney L. Millar, Gregory H. Norris, Addison Vitols, Chelsea Garcia, Samantha Seibel, Liya Anto, Christopher N. Blesso

**Affiliations:** 1 Department of Nutritional Sciences, University of Connecticut, Storrs, CT 06269, USA; courtney.millar@uconn.edu (C.L.M.); addison.vitols@uconn.edu (A.V.); chelsea.garcia@uconn.edu (C.G.); samantha.seibel@uconn.edu (S.S.); liya.anto@uconn.edu (L.A.); 2 Department of Medicine, University of Illinois, Chicago, IL 60612, USA; gnorris@uic.edu

**Keywords:** sphingolipids, eggs, atherosclerosis, phospholipids, cardiovascular, microbiota, diversity, inflammation

## Abstract

Western-style diets have been linked with dyslipidemia and inflammation, two well-known risk factors associated with cardiovascular disease (CVD). Dietary sphingomyelin (SM) has been reported to modulate gut microbiota, and lower serum lipids and inflammation in mice on Western-style diets. However, few studies have examined if nutritionally-relevant intake of dietary SM can impact atherosclerosis progression. Thus, the aim of this study was to determine if incorporating 0.1% (w/w) egg SM (ESM) (equivalent to ~750 mg/day in humans) into a high-fat (45% kcal), cholesterol-enriched diet (HFD) could prevent atheroprogression in apoE^−/−^ mice (*n* = 15/group). We found that mice fed with the ESM-rich diet had significantly lower epididymal fat mass (−46%) and tended to have higher spleen weights (+15%). There were no significant differences in serum lipids between groups. However, ESM-fed mice had significantly lower alanine aminotransferase (ALT) activity. Additionally, ESM-fed mice displayed significantly less aortic root lipid accumulation (−31%) compared to controls. This improvement in atherosclerosis was paired with over a two-fold reduction in circulating serum amyloid A (SAA) in ESM-fed mice. Finally, there was also a modulation of the gut microbiota with ESM supplementation. ESM may have the potential to prevent atherosclerosis, however further research in the clinical setting is warranted.

## 1. Introduction

Cardiovascular disease (CVD) is the leading cause of non-communicable deaths in the United States [1]. However, this disease is not confined to the U.S. alone. Globally, 35% of deaths can be attributed to CVD, reinforcing the importance of addressing the etiology of this disease [2]. Atherosclerosis is a prominent contributor of CVD, which is characterized by the accumulation of lipids, specifically cholesterol, in the arterial wall and eventually leads to lesion development [3]. 

Atherosclerosis development is enhanced by both disordered lipid metabolism and chronic inflammation. In brief, apolipoprotein(apo)-B-containing lipoproteins can become oxidized and subsequently taken up by resident macrophages in the intima of the artery. If the lipid load becomes unmanageable, foam cell formation and lesion development occur, which can result in an inflammatory plaque environment [4]. 

Diets high in some dietary lipids, such as dietary cholesterol, saturated and *trans* fats, have been shown to contribute to atherosclerosis in animal models [5,6,7,8]. Overconsumption of these dietary lipids, typically found in Western-style diets, are thought to promote these diseases through the modulation of serum lipids, inflammation, and gut microbiota [9,10,11,12], which have all been linked with atheroprogression. Thus, it is important to develop strategies to modulate the response to these lipids as a means to prevent atherosclerosis.

Diet is an attractive therapy, since modifications of diet can be easily incorporated as preventative strategies. Research has focused on functional foods, which are thought to have health-promoting benefits beyond the provision of essential nutrients [13]. Dietary bioactive compounds (DBCs) are specific components of functional foods which may be responsible for the additive benefit. A plethora of DBCs have been identified and their enrichment in diets is suggested as a strategy to prevent metabolic diseases [14]. Eggs are considered functional foods, and are a natural source of numerous DBCs, including carotenoids and bioactive proteins [15]. One other DBC of particular interest found in eggs is sphingomyelin (SM), which is a type of sphingolipid. SM consists of a ceramide molecule with a phosphorylcholine head group and is considered a zoochemical with potential health benefits [8]. Dietary sphingolipid ingestion, which includes SM, has been estimated to be approximately 300–400 mg/day in humans [16]. Much of the research concerning SM thus far has focused on potential beneficial effects of dietary SM on inhibiting intestinal lipid absorption [17], as well as preventing colon cancer [18], and NAFLD [8,19].

Our research group has previously shown that feeding dietary SM in mouse models of diet-induced obesity has promising effects in lowering circulating inflammatory markers and serum lipids [20], both of which are important for atherosclerosis development. Supplementation of high-fat diet (HFD)-fed male C57BL/6J mice with 0.1% (w/w) egg SM (ESM) for 10 weeks (human equivalent of ~400–600 mg/day) reduced serum monocyte chemoattractant protein 1 (MCP-1), an important inflammatory marker [20]. Furthermore, feeding mice with ESM also resulted in reductions in hepatic lipid accumulation and serum cholesterol [20], which may be due to the documented inhibitory effects of dietary SM on intestinal lipid absorption [21,22,23]. These previous results suggest a potential protection against atherosclerosis development, since inflammation, dyslipidemia, and hepatic lipid accumulation are all established components of its disease progression.

Recently, Chung et al. [24] reported that a relatively high-dose feeding of ESM (1.2% w/w) to apoE^−/−^ mice for 19 weeks on a chow diet led to a reduction in aortic lesion area, which was ablated with oral broad-spectrum antibiotic co-treatment. ESM is typically consumed at much lower dosages in the human diet than 1.2% w/w. Therefore, we set out to investigate the effects of a nutritionally-relevant dose of ESM (0.1% w/w) on gut microbiota composition and atherosclerosis development in apoE^−/−^ mice fed a Western-style diet. This dosage of ESM provides roughly the equivalent of consuming 750 mg/day of ESM in a 70 kg human [25]. Furthermore, ESM was fed at a 1:2 mass ratio of cholesterol in the diets, which is similar to the ratio found in egg yolk [26,27].

## 2. Materials and Methods

### 2.1. Experimental Design

In this study, 30 male apoE^−/−^ mice were obtained from Jackson Laboratory (Bar Harbor, ME, USA) at 6 weeks of age. After an acclimation period of 2 weeks, mice were randomized into two feeding groups: control diet (CTL; *n* = 15) or egg SM-containing diets (ESM; *n* = 15). Both groups were fed ad libitum a base HFD, which consisted of 45% kcal from fat and 0.2% of cholesterol (Table S1). The major fat source in the diets was palm oil, which is mainly comprised of palmitic acid and devoid of SM, which was selected in order to reduce the ingestion of SM species in the control diet. In addition to the base diet, the ESM group was supplemented with 0.1% (w/w) of purified ESM (>99%) obtained from Avanti Polar Lipids, Inc. (Alabaster, AL, USA), which is equivalent to consuming 11 mg/kg body weight per day in humans after body weight normalization [25]. Fresh food was provided twice per week and food consumption was also measured during this time. In addition, body weights were measured on a weekly basis.

After 8 weeks on their respective diets, mice were fasted for 6–8 h and blood was collected for serum and plasma isolation by cardiac puncture following ketamine/xylazine anesthesia. Prior to harvesting tissues, sterile saline was perfused into the left ventricle of the heart with the atrium clipped. The liver, spleen, intestines, and epididymal fat pads were then excised, weighed, and snap-frozen in liquid nitrogen before long-term storage at −80 °C. In addition, aortas were fixed in 10% neutral-buffered formalin for at least 48 h for en face analysis, while hearts were cut and slowly frozen in optimal cutting temperature medium and stored at −80 °C for further analysis. Mice were housed in the University of Connecticut-Storrs vivarium in a temperature-controlled room and maintained in a 14-h light/10-h dark cycle. All of the procedures proposed in this study have been approved by the Animal Care and Use Committee of the University of Connecticut-Storrs. 

### 2.2. Serum Biochemical Analysis

Serum total cholesterol, high-density lipoprotein cholesterol, non-esterified fatty acids (NEFAs) and triglycerides were measured by enzymatic kits purchased from Wako Diagnostics (Richmond, VA, Canada). Plasma glucose was measured by an enzymatic assay acquired from Pointe Scientific, Inc (Canton, MI, USA). In addition, serum alanine aminotransferase (ALT) and aspartate aminotransferase (AST) were measured by spectrophotometric methods (Pointe Scientific, Inc; Canton, MI, USA). Following kit instructions, indirect sandwich ELISAs purchased from R&D Systems (Minneapolis, MN, USA) were used to measure the inflammatory markers, interleukin-1-beta (IL-1β), MCP-1, and serum amyloid A (SAA).

### 2.3. Atherosclerosis Analysis

To evaluate aortic plaque accumulation, en face analysis was performed on the thoracic portion of aortas using previously described methods [28]. In brief, formalin-fixed aortas were opened longitudinally and stained with 0.5% oil red O (ORO) in isopropanol for plaque. A blinded observer evaluated an image of the stained aorta, by tracing the total lesion area and the total aortic area, from which a ratio was calculated.

Further analysis of atherosclerosis was evaluated using cryostat sections of the aortic root by previously described methods [28]. Following heart embedding and sectioning, formalin-fixed sections were stained with 0.5% ORO in propylene glycol and counterstained with hematoxylin and eosin. Images of the ORO-stained aortic roots were analyzed by a blinded observer, in which total lesion area (µm^2^) was measured. To evaluate the effect of ESM on connective tissue in the aortic root, slides were also stained using a Masson’s trichrome kit (Abcam; Cambridge, MA, USA). A blinded observer measured the total connective tissue area (µm^2^). 

### 2.4. RNA Isolation, cDNA Synthesis, and qRT-PCR

Hepatic, intestinal, and epididymal adipose RNA was isolated using methods previously described [29]. Glyceraldehyde-3-phosphate dehydrogenase (Gapdh), ribosomal protein, large, P0 (Rplp0), and β-actin served as internal control genes for tissue gene expression. Fold expression was calculated using the 2^−ΔΔCt^ method. The mRNA expression of genes associated with inflammation, cholesterol metabolism and bile acid synthesis was examined in livers, whereas genes associated with cholesterol absorption and markers of transintestinal cholesterol excretion were evaluated in small intestines. Table S2 lists the primer sequences used for this study. 

### 2.5. Cecal Feces Lipid Extraction

Total lipid from cecal feces samples was extracted. In brief, 4.5 mL of 95% ethanol and 0.5 mL of 50% potassium hydroxide was added to samples and incubated overnight at 50 °C. To induce phase separation, 7 mL of hexane was added. Samples underwent vigorous vortexing and centrifugation at 900× *g* for 10 min. The top lipid-laden hexane layer was aspirated and completely dried under nitrogen gas. Following any residual hexane evaporation, lipid was dissolved in 1% Triton X-100 in chloroform. Chloroform was evaporated under nitrogen gas, and the lipid–Triton mixture was re-dissolved in deionized water. Total cholesterol and free cholesterol of lipid extracts were measured by enzymatic assays per kit instructions (Wako Diagnostics; Richmond, VA, Canada). 

### 2.6. Gut Microbiota Analysis

Cecal feces samples were aseptically harvested from separately-housed mice in both the CTL and the ESM treated groups (*n* = 4–5/group), and submitted to the University of Connecticut-Storrs Microbial Analysis, Resources and Services facility for microbiota characterization using 16S V4 analysis as previously reported [30]. Following DNA extraction and amplification, PCR products were pooled for quantification, normalized, and cleaned prior to clustering into operational taxonomic unit (OTUs) at 3% variance by methods previously described [29]. Alpha diversity (within-group diversity) was assessed by Species Observed and using the Shannon diversity index. Beta diversity for dissimilarity was assessed using the Jaccard index. Data was analyzed using Permanova in R 3.3.2. Figures were drawn in R 3.3.2 using ggplot2 2.2.1. Relative abundance was calculated and figures were prepared in Excel.

### 2.7. Statistical Analysis

The differences between groups were evaluated by Student *t*-test, with *p* < 0.05 as significant. The Mann–Whitney U test was used for any data not normally distributed. All statistical analyses were conducted using GraphPad Prism version 8 software. Data are reported as mean ± SEM.

## 3. Results

### 3.1. ESM Reduces Epididymal Fat Accumulation

Body and tissue weights are shown in Table 1. There were no significant differences in body weight or food intake. However, the ESM group did have significantly lower epididymal fat after eight weeks compared to CTL, with differences in both absolute fat pad weight (−46%) and as a percentage of body weight (−44%). Despite this reduction, there were no significant differences in epididymal adipose mRNA expression for inflammatory markers between groups (Figure S1). Additionally, there were no significant differences in liver weight or food intake. ESM treatment did slightly increase both spleen weight and % spleen weight compared to the control group, however they were non-significant (*p* = 0.09 and *p* = 0.05, respectively).

### 3.2. ESM Significantly Lowers ALT Activity and Serum SAA

**Figure 1:**
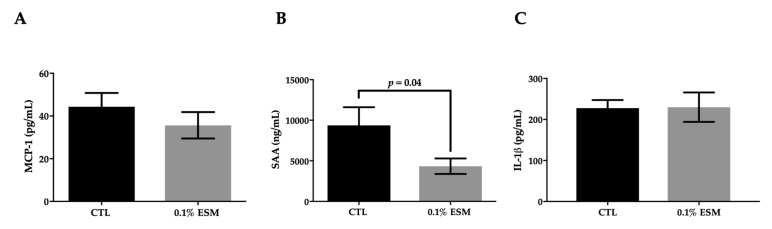
Effect of ESM on circulatory markers of inflammation. Measures of serum monocyte chemoattractant protein 1 (MCP-1) (**A**), serum amyloid A (SAA) (**B**), and interleukin (IL)-1β (**C**) after eight weeks on a high-fat diet (HFD) with or without ESM. Values are reported as mean ± SEM (*n* = 15 per group). CTL = control; ESM = egg sphingomyelin; IL-1β = interleukin-1-beta; MCP-1 = monocyte chemoattractant protein-1; SAA = serum amyloid A.

The serum biochemical analyses are shown in Table 2. There were no significant differences in serum lipids or plasma glucose. Interestingly, the ESM fed group did demonstrate a significant reduction in serum ALT activity (−22%). Further analysis revealed that supplementation with ESM lowered serum SAA concentrations (−54%), however there was no significant effect on serum MCP-1 or IL-1β concentrations (Figure 1). Although ALT was lower with ESM, there were no differences between the two groups in hepatic lipid concentrations (Figure S2A) and only weak effects on hepatic mRNA expression of both inflammatory and lipid metabolic-related genes (Figure S2B). The lipid content of the cecal feces was also not different between groups, and there were no changes in the expression of genes related to cholesterol absorption or gut-barrier proteins in the proximal small intestine (Figure S3).

### 3.3. Aortic Root Plaque Accumulation Was Lower in ESM-Fed Mice 

**Figure 2:**
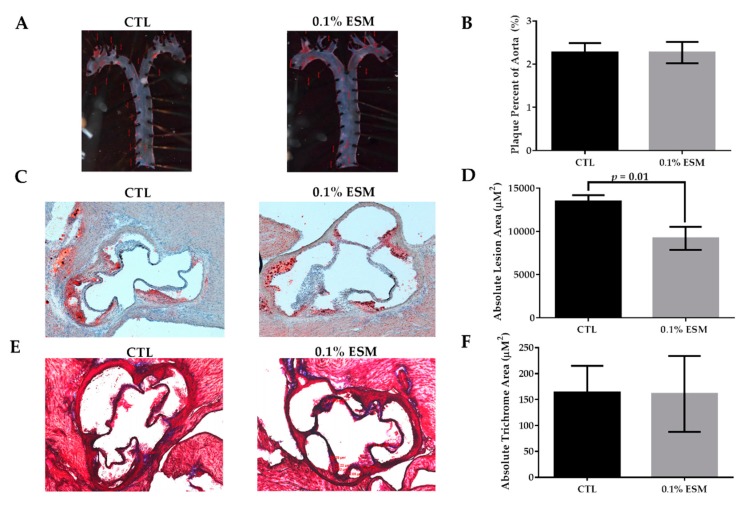
Effect of ESM on measures of atherosclerosis. Representative images of oil red O (ORO)-stained aortas from each group after eight weeks on a HFD with or without ESM (**A**) and the respective quantification of ORO-stained aortas, as percentage lesion area of the aorta (**B**). Representative images of ORO-stained aortic roots (**C**) and Masson’s trichrome stained aortic roots (**E**) from each group after 8 weeks of respective diets. Aortic root images were captured using a total of 50× magnification. Quantification of absolute lesion area (**D**) and absolute trichrome area (**F**) are shown. Values are reported as mean ± SEM (*n* = 8–9 per group).

ESM feeding had no effect on en face analysis examining ORO neutral lipid staining of the thoracic portion of aortas (Figure 2A,B), however it did significantly reduce the absolute ORO lesion area (−31%) in the aortic root (Figure 2C,D). Although neutral lipid content differed, there was no change in connective tissue content in the aortic root, as measured by trichrome stain (Figure 2E,F). Representative images of the stained aortas are shown in Figure 2A, whereas the aortic roots are shown in Figure 2C,E. 

### 3.4. Effects of ESM on Gut Microbiota Composition

**Figure 3:**
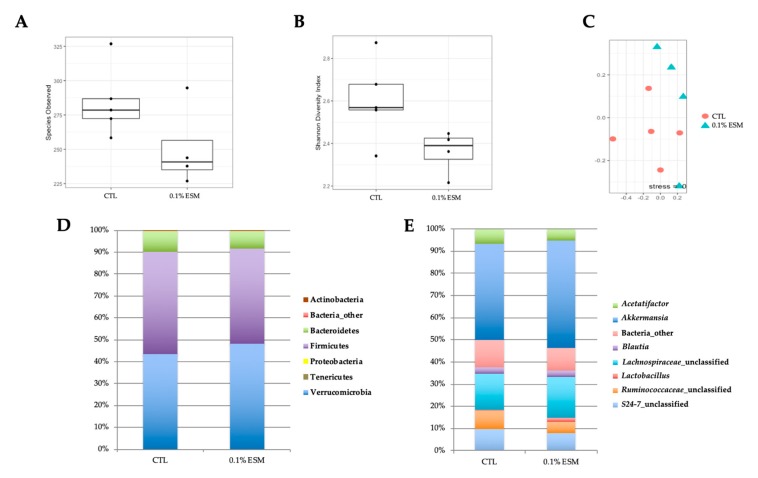
Effects of ESM on gut microbiota. Number of species observed (**A**) and the Shannon index (**B**) as measures of alpha diversity from individual mice from different cages (*n* = 4–5 per group). The Jaccard index (**C**) was calculated as a beta diversity metric (*n* = 4–5 per group). Mean relative abundance for select phyla (**D**) and genera (**E**) from both groups of mice.

There was no significant difference in species observed or the Jaccard index between groups (Figure 3A,C). However, the Shannon index, a measure of alpha diversity, tended to be lower in the ESM group compared to controls (*p* = 0.06; Figure 3B). ESM treatment significantly lowered the relative abundance of unclassified genera in the family *Ruminococcaceae* (*Ruminococcaceae*_unclassified) (8.4 ± 0.9% in CTL vs. 5.0 ± 0.6% in ESM, *p* = 0.02; Figure 3E). However, no significant differences in microbiota were observed at the phyla level. 

## 4. Discussion

Dietary SM has been previously shown to improve serum lipids and reduce systemic inflammation in C57BL/6J mice fed a HFD [29]. Dyslipidemia and inflammation are factors involved in the pathology of atherosclerosis, which is a process linked with many coronary heart diseases. In the present study, apoE^−/−^ mice were fed a HFD with added cholesterol supplemented with or without 0.1% ESM—the latter contained a mass ratio of 2:1 cholesterol/SM that mimics the ratio in egg yolk. We found that dietary ESM supplementation reduced aortic root lipid accumulation but did not change the plaque content of thoracic aorta segments measured via en face analysis. Surprisingly, this observation was paired with marginal effects on serum and hepatic lipids and hepatic gene expression. However, we did find that ESM-fed mice experienced a significant reduction in SAA and a modulation in the gut microbiota compared to controls. These findings suggest dietary ESM may be an underappreciated factor in the protection against CVD. However, clearly more research is warranted, especially in regard to the underlying mechanisms. 

There have been conflicting effects of dietary sphingolipid supplementation on serum lipids in rodent studies [19,29,31,32]. In many studies, SM has been shown to have an inhibitory effect on the intestinal absorption of luminal cholesterol [21,22,23], which has been the proposed mechanism for SM’s effect on serum lipids. Milk SM (MSM) at a dosage of 0.25% (w/w) was reported to be effective at improving both serum and hepatic lipids in HFD-fed C57BL/6J mice [29]. In the same study, 0.25% (w/w) ESM actually increased both lipid measures [29]. One explanation for the differential effects of the two sources of SM (egg vs. milk) is potentially due to MSM’s stronger inhibitory effect on luminal cholesterol absorption in rodents [8,22].

Additionally, it is hypothesized that the varying effects of dietary SM treatment is a result of the varying ratios of cholesterol/SM, as these lipids mutually inhibit each other’s intestinal absorption [29]. Rodent studies supplementing SM in diets that contained added cholesterol appeared to have more consistent, beneficial effects on serum and/or hepatic lipids [8]. As the present study did not see significant changes in serum/hepatic lipids, it is possible that benefits would have been observed with a higher dose of ESM. It is important to note that studies with SM and added cholesterol which reported a beneficial effect on lipid metabolism used higher dosages than the current study, e.g., 0.2–0.4% ESM [31] and 0.6–1.2% ESM [19]. We have previously observed reductions in serum cholesterol and hepatic lipids after 10 weeks in C57BL/6J mice fed lard-based HFDs with added cholesterol at a dose of 0.1% (w/w) ESM [20]. While numerous differences existed between this study and the current one, it appears the mouse model may be important for effects on lipid metabolism. It is possible that the apoE^−/−^ background nullified any effects on serum lipids at this intake level. Interestingly, Chung et al. [24] reported that 1.2% (w/w) ESM supplementation to a HFD (with 0.15% w/w added cholesterol) for 16 weeks also had no significant effects on serum lipids in apoE^−/−^ mice, which supports both theories that the mouse model and SM/cholesterol ratios may be important for the metabolic effects of ESM on serum/hepatic lipids. More research is warranted in this area.

Chung et al.’s [24] recent study reported that 1.2% (w/w) ESM supplementation in apoE^−/−^ mice fed a HFD for 16 weeks had no effect on en face aortic neutral lipid accumulation, while aortic root plaque content was not determined; however, on a chow diet for 19 weeks with 1.2% ESM added, there was a significant reduction in en face aortic lipid in apoE^−/−^ mice [24]. In order to consume 1.2% of SM-derived from eggs, an individual would have to eat more than 100 eggs, which is more of a pharmacological dose of ESM. Therefore, it is important to investigate whether lower intake levels, which may be more relevant to human consumption patterns, could have a similar effect. Coincidentally, the present study found a reduction in aortic root neutral lipid accumulation with 0.1% ESM in apoE^−/−^ mice on a high-fat, added cholesterol diet. Thus, these findings suggest that ESM consumption at achievable levels in a Western diet may impact atherosclerosis development. Nevertheless, it appears fat content, dosage, and duration of study, particularly in genetically-modified mouse models, are determinants of the outcomes of dietary SM and atherosclerosis. It is important to note that we did not see an effect on en face neutral lipid content of thoracic aortas, consistent with Chung et al. [24]. It is well established that, in mouse models particularly, plaque first develops in the aortic root due to the perturbed blood flow [33]. It is possible that eight weeks on a high-fat, cholesterol-enriched diet in apoE^−/−^ mice was insufficient to establish significant lesion development in the thoracic portion of the aorta. Thus, it will be important to further investigate SM supplementation not only at dosages between 0.1–1.2% (w/w) in conjunction with different levels of dietary cholesterol, but also in longer-term studies in apoE^−/−^ mice and in different atherosclerosis animal models (e.g., LDL-receptor knockout mice, hyperlipidemic rabbits). Improvements in atherosclerotic measures would not be expected to be solely dependent on changes in serum lipids. Inflammation is now well recognized as critical in the pathology of atherosclerosis [4]. Due to the importance of inflammation, future studies should investigate the effect of ESM on inflammatory cells within the atherosclerotic lesion.

In the present study, spleen weight unexpectedly tended to be larger in ESM-fed mice. Typically, spleen weight can increase with a HFD and is associated with inflammation. Future studies should explore dietary SM’s effect on splenocytes, as well as peripheral blood mononuclear cells in circulation. Additionally, we found ESM supplementation significantly reduced SAA compared to controls, however hepatic Saa1 mRNA and epididymal pro-inflammatory mRNA expression remained unchanged with ESM feeding. SAA is an acute phase protein produced mainly by the liver, which may serve as a general marker of systemic inflammation and has been linked with atheroprogression. The Women’s Ischemia Syndrome Evaluation prospective cohort study (*n* = 705) found that increased concentrations of plasma SAA was an independent predictor of future cardiovascular events in women [34]. Furthermore, SAA treatment in cell studies report an increase in LDL retention through an induction in transforming growth factor beta [35]. SAA has also been implicated in impairing the anti-inflammatory properties of high-density lipoproteins, which are critical for the body’s regulation of cholesterol via reverse cholesterol transport [36]. Future studies should look to investigate if ESM treatment acts through either of these mechanisms. 

Additionally, we found an interesting effect with ESM and liver function. In the current study, despite no difference in hepatic lipid accumulation, we did find significant reductions in ALT activity in ESM-fed mice compared to controls, suggesting protection from liver injury. ALT alone is not typically considered clinically relevant, although this coincided with reductions in circulating SAA, which is mainly derived from liver, supporting the notion that less liver injury occurred in mice fed with ESM. Future studies should perhaps utilize a SAA knockout model to determine the importance of SAA and ESM on atherosclerosis. Further, isolation of primary hepatocytes from mice treated with or without ESM could help delineate the mechanism behind these observations.

The gut microbiota has also been correlated with atherosclerosis development. Previous studies in mice have linked dietary SM with changes in gut microbiota [29,30,37] and/or effects dependent on gut microbiota [24]. Gut dysbiosis can allow for endotoxins and/or whole bacteria to translocate and induce systemic inflammation [38]. In this study, we found no significant effect on microbial diversity of the gut. However, we found that the Shannon index, a measure of species richness, tended to be lower with ESM feeding (*p* = 0.06). Additionally, there was a significant reduction in *Ruminococcaceae* in the cecal feces of ESM-fed mice. Interestingly, a depletion of fecal *Ruminococcaceae* was found in heart failure patients compared to control patients [39], which does not support our results. Therefore, the modest changes in gut microbiota do not appear to explain differences in aortic root plaque observed in mice fed ESM in the current study. Mice and humans have different compositions of gut microbiota, which may be the reason for the opposing effects. Furthermore, gut microbes are part of a complex microenvironment of the gut and evidence linking diet, gut microbiota, and disease states are still relatively associative in nature. Previous studies in C57BL/6J mice demonstrating bifidogenic effects of diets containing 0.25% (w/w) milk SM [29,37] were not supported by the current study with a lower dose of ESM; however, the current study is in agreement with what has been reported in mice fed with 0.1% (w/w) milk SM [30]. It is possible that the reduced Shannon index and *Ruminococcaceae* are related to the expected inhibition of dietary lipid absorption by ESM. Although, it is important to note that the cecal fecal lipids, which are a measure of lipid excretion, were not significantly different. Future studies should investigate nutritionally-relevant doses of ESM with or without antibiotics to determine the importance of its effect on the microbiota and atherosclerosis development, as well as further investigating colonic inflammation. 

## 5. Conclusions

Overall, we observed a reduction in aortic neutral lipid accumulation in ESM-supplemented apoE^−/−^ mice fed a HFD with added cholesterol, which was paired with a reduction in circulating SAA, a general marker of systemic inflammation. There were no significant effects on serum or hepatic lipids, as well as hepatic gene expression. Additionally, there was a modest modulation of gut microbiota by ESM. Thus, a lowering of inflammation may have contributed to the attenuation in aortic root atherosclerosis observed in the current study. Overall, this study does not fully elucidate the mechanism for ESM’s effect on atherosclerosis and further research is required. Importantly, ESM was fed at a 1:2 mass ratio of cholesterol in the diets, which is similar to the ratio found in egg yolk, suggesting it may be an important factor in the egg yolk food matrix that may confer health benefits and/or counteract effects of dietary cholesterol. However, future studies are warranted to expand on these findings.

## Figures and Tables

**Table 1:** Weights and Food Intake.

Measure	Control	0.1% ESM	*p*-Value
Body Weight (g)	29.6 ± 0.34	28.8 ± 0.51	0.16
Liver Weight (g)	1.32 ± 0.05	1.26 ± 0.05	0.43
% Liver Weight	4.55 ± 0.12	4.49 ± 0.12	0.75
Epididymal Fat Weight (g)	0.46 ± 0.07	0.25 ± 0.03	0.01
% Epididymal Weight	1.60 ± 0.24	0.90 ± 0.08	0.01
Spleen Weight (g)	0.13 ± 0.01	0.15 ± 0.01	0.09
% Spleen Weight	0.46 ± 0.02	0.53 ± 0.03	0.05
Foot Intake (g/mouse)	3.18 ± 0.07	3.25 ± 0.08	0.51

Values are mean ± SEM for each group (*n* = 15/group). Student *t*-tests were performed to detect statistical differences with *p* < 0.05. ESM = egg sphingomyelin.

**Table 2:** Serum Biochemical Analysis.

Measure	Control	0.1% ESM	*p*-Value
Total Cholesterol (mg/dL)	797 ± 33.8	744 ± 47.4	0.36
HDL-C (mg/dL)	78.5 ± 9.8	63.3 ± 12.9	0.35
Non-HDL-C (mg/dL)	719 ± 34.8	681 ± 49.6	0.53
Triglycerides (mg/dL)	86.9 ± 10.0	87.6 ± 13.6	0.97
NEFAs (mg/dL)	0.49 ± 0.05	0.47 ± 0.05	0.71
Glucose (mg/dL)	269 ± 19.6	288 ± 33.8	0.13
ALT (U/L)	86.5 ± 7.0	67.0 ± 4.6	0.03
AST (U/L)	104 ± 8.8	88.1 ± 6.5	0.16

Values are mean ± SEM for each group (*n* = 15/group). Student *t*-tests were performed to detect statistical differences with *p* < 0.05. ALT = alanine aminotransferase; AST = aspartate aminotransferase; HDL-C = high-density lipoprotein cholesterol; NEFAs = non-esterified fatty acids.

## Supplementary Materials

The following are available online at https://www.mdpi.com/2072-6643/11/5/1124/s1, Figure S1: Effect of ESM on epididymal adipose gene expression, Figure S2: Effect of ESM on liver lipids and hepatic gene expression, Figure S3: Effect of ESM on cecal feces lipids and intestinal gene expression, Table S1: Composition of diets, Table S2: Primer List for qRT-PCR.
