# Supplementary material for: Dietary Egg Sphingomyelin Prevents Aortic Root Plaque Accumulation in Apolipoprotein-E Knockout Mice
Source: Nutrients. 2019 May 21;11(5):1124. doi: 10.3390/nu11051124 (PMC6566691; doi:10.3390/nu11051124)
Supplement: Supplementary file 1 [file nutrients-11-01124-s001.zip › Supplemental/Supplementary Figure 1.docx]

**Figure S1.** Effect of ESM on epididymal adipose gene expression. Gene expression in the epididymal fat pad for inflammatory markers (*n* = 9 per group). Values are reported as mean ± SEM.
