# Supplementary material for: Dietary Egg Sphingomyelin Prevents Aortic Root Plaque Accumulation in Apolipoprotein-E Knockout Mice
Source: Nutrients. 2019 May 21;11(5):1124. doi: 10.3390/nu11051124 (PMC6566691; doi:10.3390/nu11051124)
Supplement: Supplementary file 1 [file nutrients-11-01124-s001.zip › Supplemental/Supplementary Figure 2.docx]

**
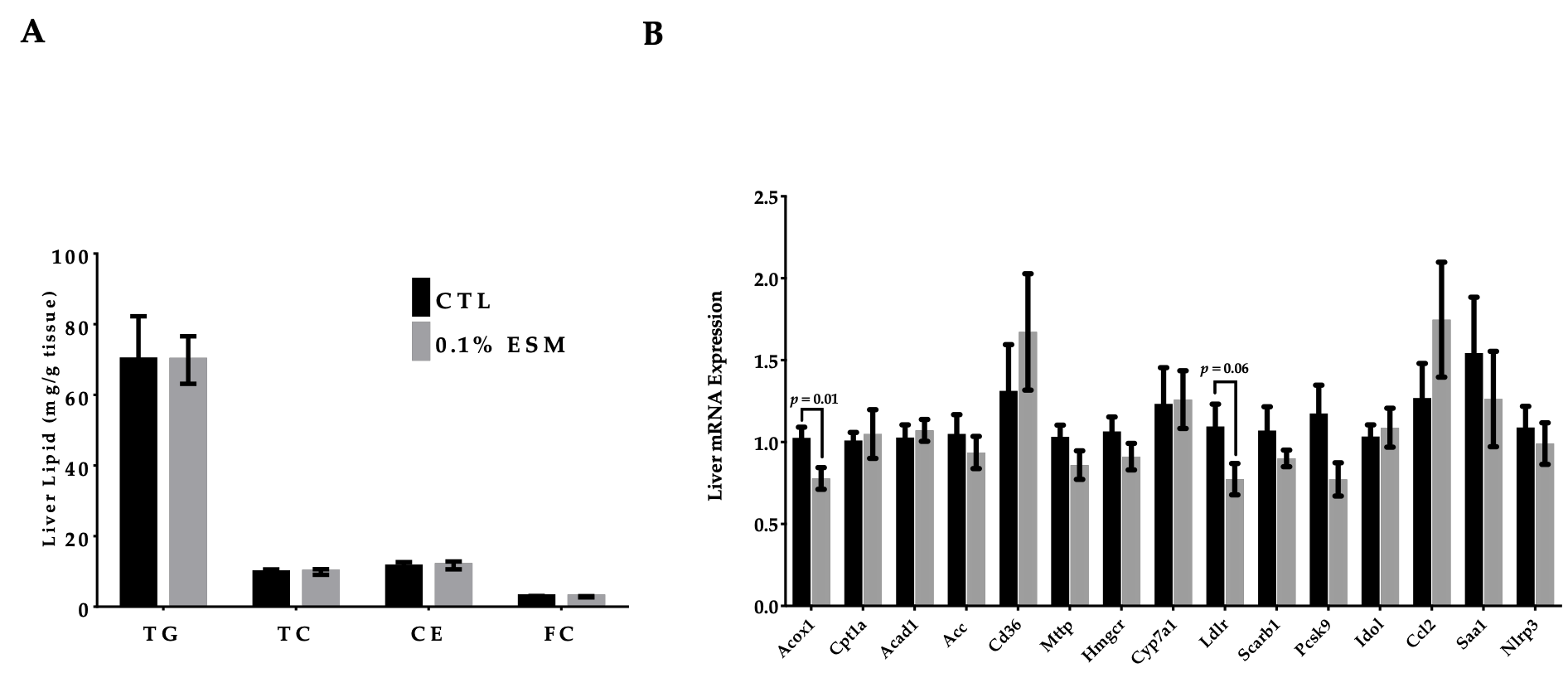
**

**Figure S2.** Effect of ESM on liver lipids and hepatic gene expression. Hepatic lipid levels in mice on HFD with or without ESM for 8 weeks (A). Gene expression from liver tissue for lipid metabolism and inflammatory genes (B) after 8 weeks diets. Values are reported as mean ± SEM (*n* = 10 per group). TG, triglycerides; TC, total cholesterol; CE, cholesteryl esters; FC, free cholesterol.
