# Supplementary material for: Dietary Egg Sphingomyelin Prevents Aortic Root Plaque Accumulation in Apolipoprotein-E Knockout Mice
Source: Nutrients. 2019 May 21;11(5):1124. doi: 10.3390/nu11051124 (PMC6566691; doi:10.3390/nu11051124)
Supplement: Supplementary file 1 [file nutrients-11-01124-s001.zip › Supplemental/Supplementary Figure 3.docx]

**Figure S3.** Effect of ESM on cecal feces lipids and intestinal gene expression. Cecal feces lipid levels in mice on HFD with or without ESM for 8 weeks (A; *n* = 9 per group). Gene expression in the proximal small intestine for lipid metabolism and gut barrier-related genes (B; *n* = 15 per group). Values are reported as mean ± SEM. TC, total cholesterol; FC, free cholesterol.
