# Supplementary material for: Dietary Egg Sphingomyelin Prevents Aortic Root Plaque Accumulation in Apolipoprotein-E Knockout Mice
Source: Nutrients. 2019 May 21;11(5):1124. doi: 10.3390/nu11051124 (PMC6566691; doi:10.3390/nu11051124)
Supplement: Supplementary file 1 [file nutrients-11-01124-s001.zip › Supplemental/Supplementary Table 1.docx]

**Table S1. Composition of diets**

| **Diet Component** | | **Control (g/kg)** | | **0.1% ESM (g/kg)** | |
| --- | --- | --- | --- | --- | --- |
| Casein | 195 | | 195 | |  |
| L-Cystine | 3 | | 3 | |  |
| Sucrose | 340 | | 340 | |  |
| Corn Starch | 54.96 | | 54.96 | |  |
| Maltodextrin | 60 | | 59 | |  |
| Palm Oil | 210 | | 210 | |  |
| Soybean Oil | 20 | | 20 | |  |
| Cellulose | 50 | | 50 | |  |
| Mineral Mix, AIN-93-MX | 43 | | 43 | |  |
| Vitamin Mix, AIM-93-VX | 19 | | 19 | |  |
| Choline Bitartrate | 3 | | 3 | |  |
| TBHQ | 0.04 | | 0.04 | |  |
| Cholesterol | 2 | | 2 | |  |
| Egg Sphingomyelin | 0 | | 1 | |  |
|  |  | |  | |  |
