# Supplementary material for: Dietary Egg Sphingomyelin Prevents Aortic Root Plaque Accumulation in Apolipoprotein-E Knockout Mice
Source: Nutrients. 2019 May 21;11(5):1124. doi: 10.3390/nu11051124 (PMC6566691; doi:10.3390/nu11051124)
Supplement: Supplementary file 1 [file nutrients-11-01124-s001.zip › Supplemental/Supplementary Table 2.docx]

**Table S2.** Primer List for qRT-PCR

| **Gene** | **Protein*** | **5'-Forward Primer-3'** | **5'-Reverse Primer-3'** |
| --- | --- | --- | --- |
| *Abcg5* | ABCG5 | CGTGGCGGACCAAATGA | GCTCGCCACTGGAAATTCC |
| *Actb* | β-Actin | TCCTTCTTGGGTATGGAATCCT | CAGCACTGTGTTGGCATAGA |
| *Acad1* | ACAD | GGCTTGCTTGGCATCAACA | AGAGCAAGTCCCCACCAATG |
| *Acc* | ACC | CGCTCAGGTCACCAAAAAGAAT | GTCCCGGCCACATAACTGAT |
| *Acox1* | ACOX | CCCAAGACCCAAGAGTTCATTC | CAGGCCACCACTTGATGGA |
| *AdipoQ* | ADIPOQ | CAACCAACAGAATCATTATG | GGTAAGAGAAGTAGTAGAGT |
| *Ccl2* | CCL2 | TTCCTCCACCACCATGCAG | CCAGCCGGCAACTGTGA |
| *Cd36* | CD36 | CTTACACATACAGAGTTCGTTATC | TCCAACAGACAGTGAAGG |
| *Cpt1a* | CPT1 | GAACCCCAACATCCCCAAAC | TCCTGGCATTCTCCTGGAAT |
| *Cyp7a1* | CYP7A1 | AGCAACTAAACAACCTGCCAGTACTA | GTCCGGATATTCAAGGATGCA |
| *F4/80* | F4/80 | GAGTGGAATGTCAAGATGTTA | CAGTGGAAGAAGAGAAGC |
| *Fabp2* | FABP2 | GTCTAGCAGACGACGGAACGGA | AGAAACCTCTCGGACAGCAA |
| *Gapdh* | GAPDH | TGTGTCCGTCGTGGATCTGA | CCTGCTTCACCACCTTCTTGAT |
| *Hmgcr* | HMGCR | GATGATTATGTCTTTAGGCTTG | CAGAGAGAAACACTTGGT |
| *Idol* | IDOL | AGGAGATCAACTCCACCTTCTG | ATCTGCAGACCGGACAGG |
| *Ldlr* | LDL-R | TTCCTGTCCATCTTCTTCC | GACCATCTGTCTTGAGGG |
| *Mttp* | MTTP | CTACCAGGCCCAACAAGAC | CGCTCAATTTTGCATGTATCC |
| *Nlrp3* | NLRP3 | AGAGCCTACACTTCGGTGAAATG | CCACGCCTACCAGGAAATCTC |
| *Npc1l1* | NPC1L1 | CGTCTGTCCCCGCCTATACA | CTAATGACACCAGCTGCTTGGT |
| *Ocln* | OCLN | CACCTATCACTTCAGATCAACAA | CAGCAGCCATGTACTCTTC |
| *Pcsk9* | PCSK9 | AGGTGGAGGTGTATCTCTTAGA | GGCTATGTCATCAAGGTTCTA |
| *Rplp0* | RPLP0 | CCTGAAGTGCTCGACATCAC | CCACAGACAATGCCAGGAC |
| *Saa1* | SAA | CCAATTACTACAGACCTCCT | AGCATCTTCAGTGTTCCTA |
| *Scarb1* | SRB1 | CGGACTCAGCAAGATCGA | CGAGGATTCGGGTGTCAT |
| *Tjp1* | TJP1 | AGAGGAAGAGCGAATGTC | TTCGGTTCTGGAAGAGTG |
| *Tnf* | TNF-α | GGCTGCCCCGACTACGT | ACTTTCTCCTGGTATGAGATAGCAAAT |

*Abbreviations used: ABCG5, ATP Binding Cassette Subfamily G Member 5; β-actin, beta-actin; ACAD, acyl-CoA dehydrogenase; ACC, acetyl-CoA carboxylase; ACOX, acyl-CoA oxidase 1; ADIPOQ, adiponectin; CCL2, C-C motif chemokine ligand 2; CD36, cluster of differentiation 36; CPT1, carnitine palmitoyltransferase 1A; CYP7A1, cholesterol 7 alpha-hydroxylase 1; F4/80, ﻿EGF-like module-containing mucin-like hormone receptor-like 1; FABP2, fatty acid binding protein 2; GAPDH, glyceraldehyde 3-phosphate dehydrogenase; HMGCR, 3-hydroxy-3-methyl-glutaryl-coenzyme A reductase; IDOL, inducible degrader of low-density lipoprotein receptor ; LDL-R, low-density lipoprotein receptor; MTTP, microsomal triglyceride transfer protein; NLRP3, Nucleotide-binding domain, leucine-rich-containing family, pyrin domain-containing-3; NPC1L1, Niemann-Pick C1-Like 1; OCLN, occludin; PCSK9, pro-protein convertase subtilisin/kexin type 9; RPLP0, Ribosomal Protein Lateral Stalk Subunit P0; SAA, serum amyloid A; SRB1, scavenger receptor class B member 1;TJP1, Tight Junction Protein ZO-1; TNF-α, tumor necrosis factor alpha.
